# No evidence for an association of voxel-based morphometry with short-term non-motor outcomes in deep brain stimulation for Parkinson’s disease

**DOI:** 10.1038/s41531-024-00695-1

**Published:** 2024-04-26

**Authors:** Philipp Alexander Loehrer, Wibke Schumacher, Stefanie T. Jost, Monty Silverdale, Jan Niklas Petry-Schmelzer, Anna Sauerbier, Alexandra Gronostay, Veerle Visser-Vandewalle, Gereon R. Fink, Julian Evans, Max Krause, Alexandra Rizos, Angelo Antonini, Keyoumars Ashkan, Pablo Martinez-Martin, Christian Gaser, K. Ray Chaudhuri, Lars Timmermann, Juan Carlos Baldermann, Haidar S. Dafsari, On behalf of EUROPAR and the International Parkinson and Movement Disorders Society Non-Motor Parkinson’s Disease Study Group

**Affiliations:** 1 Department of Neurology, Philipps-University Marburg, Marburg, Germany; 2 University of Cologne, Faculty of Medicine and University Hospital Cologne, Department of Neurology, Cologne, Germany; 3 University of Cologne, Faculty of Medicine and University Hospital Cologne, Department of Pediatrics, Cologne, Germany; 4 Department of Neurology and Neurosurgery, Salford Royal Foundation Trust, Greater Manchester, United Kingdom; 5 National Parkinson Foundation Centre of Excellence, King’s College Hospital, London, United Kingdom; 6 University of Cologne, Faculty of Medicine and University Hospital Cologne, Department of Stereotactic and Functional Neurosurgery, Cologne, Germany; 7 Cognitive Neuroscience, Institute of Neuroscience and Medicine (INM-3), Research Center Jülich, Jülich, Germany; 8 Department of Neurology and Neurosurgery, Salford Royal NHS Foundation Trust, Manchester Academic Health Science Centre, University of Manchester, Greater Manchester, United Kingdom; 9 University of Cologne, Faculty of Medicine and University Hospital Cologne, Department of Radiation Oncology, Cyberknife Center, Cologne, Germany; 10 Department of Neuroscience, University of Padua, Padua, Italy; 11 Department of Neurosurgery, King’s College Hospital, London, United Kingdom; 12 Center for Networked Biomedical Research in Neurodegenerative Diseases (CIBERNED), Carlos III Institute of Health, Madrid, Spain; 13 Department of Psychiatry and Psychotherapy, Jena University Hospital, Jena, Germany; 14 Department of Neurology, Jena University Hospital, Jena, Germany; 15 German Center for Mental Health (DZPG), Site Jena-Magdeburg-Halle, Germany; 16 Center for Intervention and Research on adaptive and maladaptive brain Circuits underlying mental health (C-I-R-C), Jena-Magdeburg-Halle, Germany; 17 The Maurice Wohl Clinical Neuroscience Institute, King’s College London, London, United Kingdom; 18 Klinik für Psychiatrie und Psychotherapie, Universitätsklinikum Freiburg, Freiburg im Breisgau, Germany

**Keywords:** Parkinson's disease, Neuroscience, Predictive markers

## Abstract

Deep brain stimulation of the subthalamic nucleus (STN-DBS) is an established therapy in advanced Parkinson’s disease (PD). Motor and non-motor outcomes, however, show considerable inter-individual variability. Preoperative morphometry-based metrics have recently received increasing attention to explain treatment effects. As evidence for the prediction of non-motor outcomes is limited, we sought to investigate the association between metrics of voxel-based morphometry and short-term non-motor outcomes following STN-DBS in this prospective open-label study. Forty-nine PD patients underwent structural MRI and a comprehensive clinical assessment at preoperative baseline and 6-month follow-up. Voxel-based morphometry was used to assess associations between cerebral volume and non-motor outcomes corrected for multiple comparisons using a permutation-based approach. We replicated existing results associating volume loss of the superior frontal cortex with subpar motor outcomes. Overall non-motor burden, however, was not significantly associated with morphometric features, limiting its use as a marker to inform patient selection and holistic preoperative counselling.

## Introduction

Deep brain stimulation (DBS) of the subthalamic nucleus (STN) is an established therapy for the treatment of motor and non-motor symptoms in advanced Parkinson’s disease (PD)^1–3^. Despite its well-established effects at the group level, individual symptom relief varies significantly, complicating preoperative patient selection and counseling^4^. To predict outcomes and support preoperative management, neuroimaging-based biomarkers using advanced imaging technologies, such as tractography and functional MRI, have proven useful^4,5^. Their widespread clinical application, however, is limited by the need for additional and sometimes time-consuming scanning protocols and the expertise to analyse and translate their results^6^. Therefore, the association of postoperative outcomes with metrics based on T1-weighted sequences obtained in clinical routine during surgical planning has been investigated. Particularly, analyses focussing on morphometric tissue features, such as voxel-based morphometry (VBM), have lately received increasing attention to monitor clinical progression and treatment effects^6^. In a recent meta-analysis including 1253 patients enrolled in 24 studies, Wang and colleagues identified specific areas whose morphometric features were associated with outcomes following STN-DBS^6^. Here, atrophy of the motor cortex and thalamus was associated with below-average improvement in motor symptoms. On the other hand, outcome prediction of non-motor symptoms has received little attention, with studies focusing on cognitive decline and immediate psychiatric alterations such as postoperative confusion, delirium, and impulsivity^6^. Poor outcomes in verbal memory were associated with hippocampal atrophy at baseline, while immediate psychiatric complications were related to caudal middle frontal cortex atrophy^6^. As STN-DBS is associated with beneficial short-term outcomes in a range of non-motor symptoms such as sleep/fatigue, attention/memory, and mood/apathy^7,8^, in the present study, we sought to explore the association between overall non-motor symptom burden and volumetric properties.

## Results

### Clinical outcomes

**Fig. 1:**
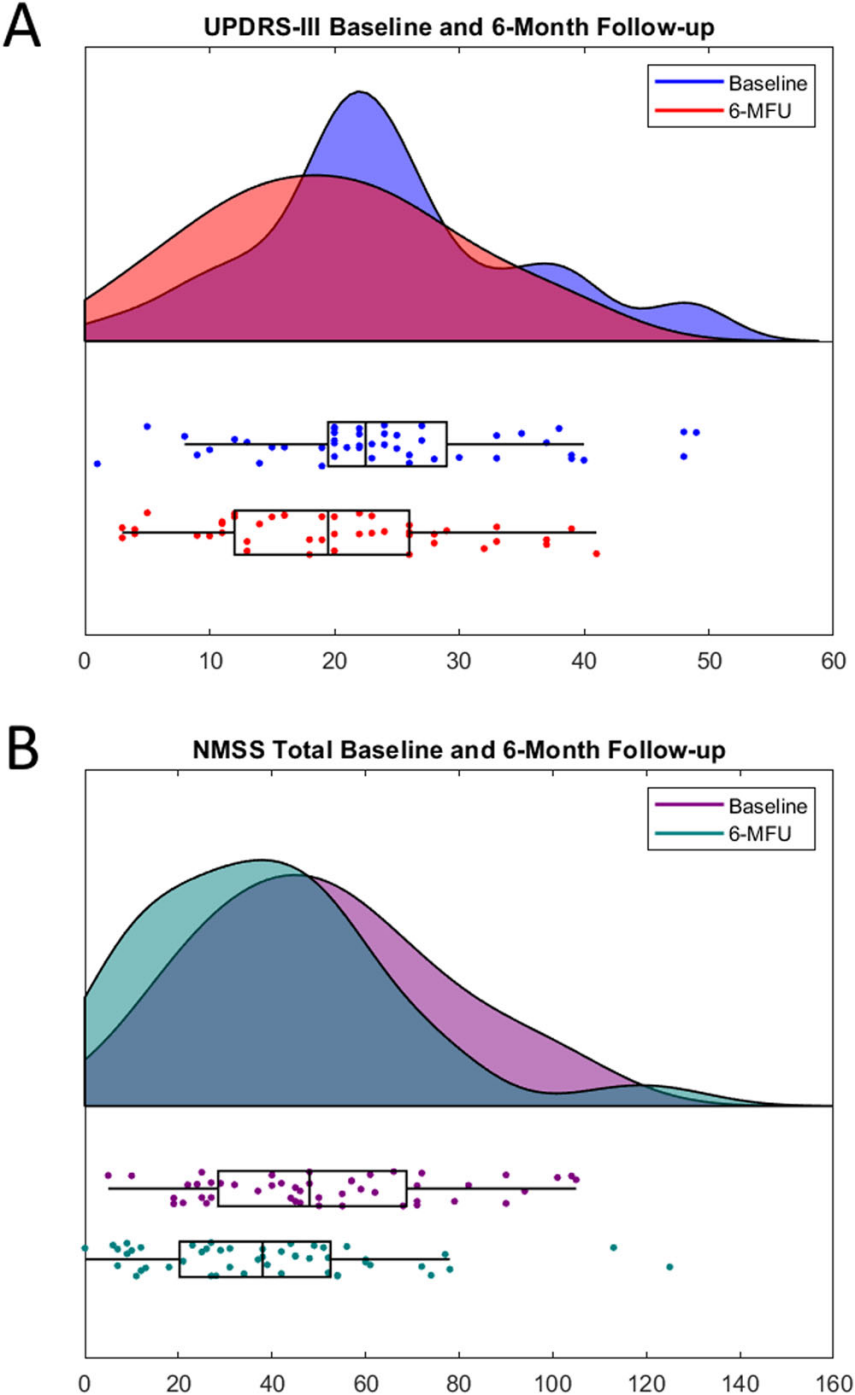
Visualization of baseline and 6-month follow-up values of clincial variables. Visualization of baseline and 6-month follow-up values of the unified Parkinson’s disease rating scale part III (**A**) and non-motor symptom scale-total score (**B**). Center line indicates the median, box limits represent upper and lower quartiles, and whiskers indicate the most extreme data points not considered outliers.

Forty-nine patients with PD (31 males, mean age 64.5 ± 8.2 years) were enrolled. At the 6-month follow-up, the following scales improved: NMSS-total score (false discovery rate (FDR) correction applied, *p* = 0.006, Cohen’s *d* = 0.46; evaluating the global non-motor symptom burden), PDQ-8 SI (FDR *p* < 0.001, Cohen’s *d* = 0.52; evaluating quality of life), UPDRS-III (FDR *p* = 0.023, Cohen’s *d* = 0.45; evaluating motor symptom severity), SCOPA-M activities of daily living (FDR *p* < 0.001, Cohen’s *d* = 0.56; evaluating activities of daily living), SCOPA-M motor complications (FDR *p* < 0.001, Cohen’s *d* = 0.85; evaluating motor complications), and LEDD (FDR *p* < 0.001, Cohen’s *d* = 1.06; evaluating the total daily dose of levodopa). Analysis of NMSS-domains revealed beneficial effects of STN-DBS on sleep/fatigue (*p* = 0.003, Cohen’s *d* = 0.55), perceptual problems/hallucinations (*p* = 0.023, Cohen’s *d* = 0.39), urinary symptoms (*p* = 0.023, Cohen’s *d* = 0.35), and miscellaneous symptoms (*p* < 0.001, Cohen’s *d* = 0.68). Longitudinal changes in clinical outcomes are reported in Table 1 and displayed in Fig. 1.

**Table 1:** Baseline characteristics and outcomes at baseline and 6-month follow-up

	*N*	*M*	SD					
Age [y]	49	64.47	8.23					
Disease duration [y]	49	9.99	4.35					
Sex (female/male) [%]	49	18/31	[36.7/63.3]					

	Baseline			6-MFU			Baseline vs. 6-MFU	
	*n*	*M*	SD	*n*	*M*	SD	*p*	Cohen’s *d*
NMSS-total score	49	51.1	25.2	49	39.2	26.3	**0.003**	**0.46**
Cardiovascular	49	1.8	3.0	49	1.4	2.0	0.656	0.13
Sleep/fatigue	49	13.2	9.1	49	9.0	5.9	**0.001**	**0.55**
Mood/apathy	49	5.3	5.7	49	6.6	12.1	0.409	−0.13
Perceptual problems /hallucinations	49	1.9	3.9	49	0.7	2.1	**0.012**	**0.39**
Attention/memory	49	4.6	4.8	49	3.7	4.1	0.201	0.20
Gastrointestinal	49	4.3	4.6	49	5.0	5.8	0.733	−0.12
Urinary	49	9.8	8.9	49	6.9	7.2	**0.015**	**0.35**
Sexual function	49	2.3	4.0	49	1.6	2.9	0.111	0.21
Miscellaneous	49	8.1	6.5	49	4.4	4.0	**<0.001**	**0.68**
PDQ-8 SI	43	33.0	16.3	48	24.9	14.5	**<0.001**	**0.52**
UPDRS-III	49	24.4	10.7	45	19.7	10.0	**0.014**	**0.45**
SCOPA-M activities of daily living	49	8.0	2.7	49	6.3	3.2	**<0.001**	**0.56**
SCOPA-M motor complications	49	4.0	2.8	45	1.8	2.3	**<0.001**	**0.85**
LEDD [mg]	49	1102	590.3	48	588.6	341.9	**<0.001**	**1.06**

Demographic characteristics and outcome parameters at baseline and 6-month follow-up. Reported *p* values are corrected for multiple comparisons using Benjamini–Hochberg’s method (six main outcome scales). Bold font highlights significant results, *p* < 0.05.

*6-MFU* 6-month follow-up, *LEDD* levodopa equivalent daily dose, *LEDD-DA* LEDD of dopamine agonists, *NMSS* non-motor symptom scale, *PDQ-8 SI* 8-item Parkinson’s disease questionnaire summary index, *SCOPA-M* scales for outcomes in Parkinson’s disease-motor, *SD* standard deviation, *UPDRS-III* unified Parkinson’s disease rating scale part III.

### Association of voxel-based morphometry metrics and postoperative motor symptom change

**Fig. 2:**
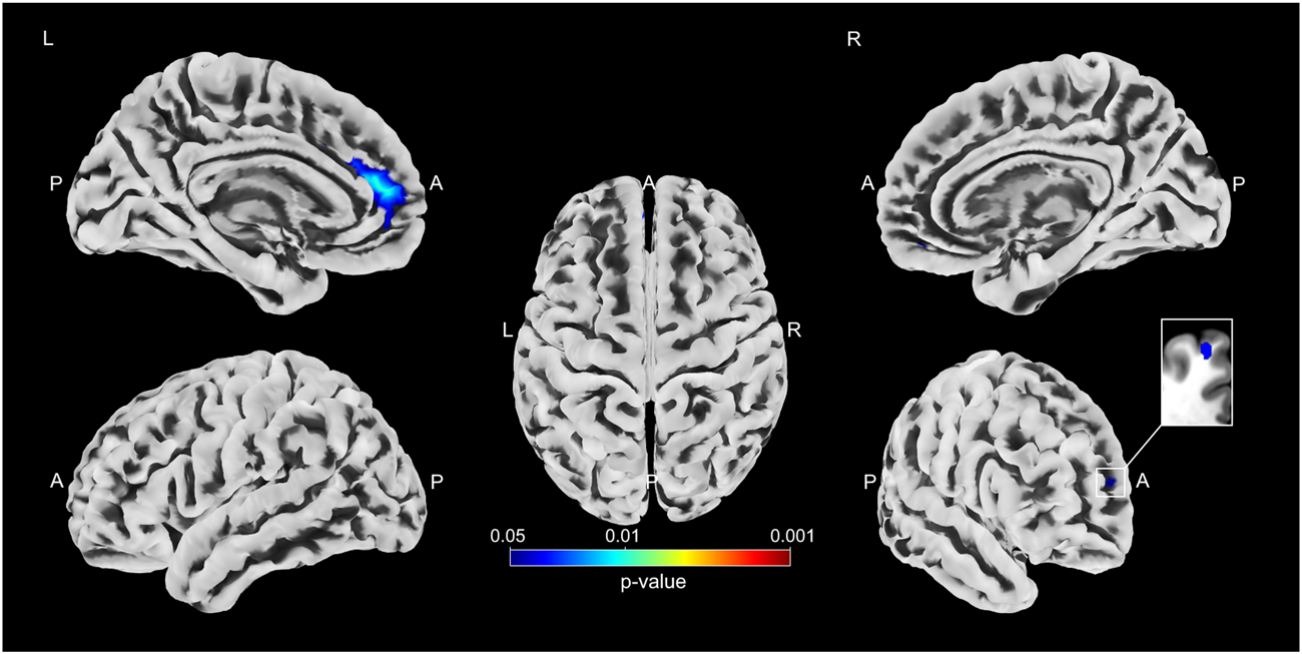
Clusters with an association between metrics of voxel-based morphometry and postoperative change in UPDRS-III as revealed by the whole brain analysis. Results are displayed as surface overlays (please see Supplementary Fig. 1 for a result display on axial slices). Clusters denote regions of low cortical volumes significantly associated with poor motor response to deep brain stimulation. *P* values were corrected for multiple comparisons using a permutation-based approach and thresholded at *p* < 0.05, family-wise error-corrected.

A multiple regression analysis assessed the relationship between motor response to STN-DBS and VBM metrics. Following a threshold-free cluster enhancement (TFCE) approach^9^ to correct for multiple comparisons, a cluster within the bilateral superior frontal cortex showed an association with changes in postoperative motor function, i.e., lower volumes were associated with poor postoperative outcomes (left superior frontal cortex: *p* = 0.008, right superior frontal cortex: *p* = 0.045; c.f. Fig. 2, Supplementary Fig. 1, and Table 2). In a complementary analysis, we used ComBat to harmonize VBM metrics across scanners used in the present study and reran the regression analysis on the harmonized values incorporating analysis of covariance (ANCOVA) to control for baseline inhomogeneity and disease duration as an additional covariate. Following the TFCE-approach the results remained consistent, when applied to data that incorporated an ANCOVA (left superior frontal cortex: *p* = 0.003, right superior frontal cortex: *p* = 0.029; c.f. Supplementary Table 1). After incorporating an ANCOVA and accounting for the covariate disease duration, the left superior frontal cortex remained associated with changes in postoperative motor function (*p* = 0.005), while there was a trend towards significance for the cluster in the right superior frontal cortex (*p* = 0.054; c.f. Supplementary Table 2).

**Table 2:** Association between brain morphometry and UPDRS-III

Cluster	Location	*p* value	Cluster size	MNI152-coordinates		
				X	Y	Z
1	Left superior frontal cortex	0.008	1069	−14	50	18
2	Right superior frontal cortex	0.045	66	12	59	15

Characteristics of clusters with an association between metrics of voxel-based morphometry and postoperative change in UPDRS-III. “Cluster” denotes clusters with a significant association between low cortical volumes and poor motor response to deep brain stimulation (DBS). “Location” indicates the anatomical landmark comprising the majority of voxels of a cluster, according to the Desikan–Killiany Atlas. *P* Values are clusterwise *p* values corrected for multiple comparisons. Cluster size denotes the extent of a cluster in a voxel. “MNI152-coordinates” describe the coordinates of the cluster’s center of gravity in MNI152-space.

### Association of voxel-based morphometry metrics and postoperative non-motor symptom change

No significant associations were observed between VBM metrics and change scores of NMSS-total score (NMSS-T) and NMSS-domains, following a TFCE-approach to correct for multiple comparisons. This effect remained even after harmonization with ComBat and incorporating ANCOVA to control for baseline inhomogeneity and disease duration as a covariate.

### Association of voxel-based morphometry metrics and postoperative motor and non-motor symptom change in a Bayes factor mapping approach

In a complementary analysis, we mapped neural correlates of both motor and non-motor outcomes using Bayes factor mapping by Bayesian general linear models on the harmonized data. Clusters in the bilateral cerebellum, as well as the left superior frontal cortex and the left superior parietal cortex, showed very strong evidence for an association with postoperative motor outcomes (c.f. Supplementary Fig. 2 for thresholded and Supplementary Fig. 4 for unthresholded results). While clusters in the bilateral hippocampus, as well as the left insular cortex, showed very strong evidence for an association with postoperative non-motor outcomes (c.f. Supplementary Fig. 3 for thresholded and Supplementary Fig. 5 for unthresholded results).

## Discussion

In the present study, we investigated the association between brain morphometric features and changes in clinical outcomes following STN-DBS in PD. We replicated findings that impaired integrity of the frontal cortex is associated with subpar improvement in motor symptoms following STN-DBS. In contrast, changes in non-motor symptoms were not significantly associated with features of brain morphometry using a frequentist approach.

Employing a whole brain voxel-based morphometry analysis, we identified an association between reduced volume of the bilateral superior frontal cortex and poor motor outcomes after neurostimulation in PD. Specifically, the left cluster was located in the medial aspect of the left hemisphere, partially overlapping with areas of the anterior cingulate cortex. In contrast, the cluster in the right hemisphere was situated more laterally in an area that overlapped with the medial prefrontal cortex. Despite these variations, both clusters were located in the superior frontal cortex, according to the Desikan–Killiany Atlas. This finding is consistent with previous studies demonstrating an association between poor motor outcomes and reduced cortical thickness and a diminished volume of this region^10,11^. Importantly, the superior frontal cortex comprises the critical structures of the motor network for movement generation and control^10,12,13^. Information processing within this network is altered in PD and modulated by dopaminergic replacement therapy and DBS, resulting in improved motor function^14,15^. In STN-DBS, this modulation is in part elicited by antidromic conveyance of stimulation signals via the hyperdirect pathway that directly links the subthalamic nucleus to structures of the motor network^16^. Consequently, the integrity of the frontal cortex seems crucial for STN-DBS to exert its effects and might serve as a marker to predict motor response after DBS surgery. In future studies, larger sample sizes in multicentre cohorts are needed to define patient-specific thresholds and thus implement bilateral superior frontal cortex volume as a biomarker for individual outcome prediction.

Contrary to our expectations, there were no significant associations between morphometric features and postoperative changes in non-motor symptoms using a frequentist approach with null hypothesis significance testing. When employing Bayesian statistics, however, we could identify regions in the bilateral hippocampus and left insula which showed evidence for an association with postoperative non-motor outcomes. Several considerations have to be taken into account when interpreting the disparity in the present findings. First, there are methodological differences in the two approaches that potentially contribute to the present findings. Frequentist methods typically use *p* values and control the Type I error rate, while Bayesian methods use Bayes factors, which quantify evidence for or against a hypothesis.

Second, it is important to note that, in general, Bayes factor mapping tends to be more liberal than frequentist approaches with null hypothesis testing, which contributes to the discrepancies in the results of the present study^17^. Third, while Bayes factor mapping can be advantageous to apply in situations where effect sizes and clusters associated with outcomes are small, there tends to be a pronounced overshoot of evidence for h1 with many false positives in situations with large effect sizes or samples^17^. Fourth, Bayes factor mapping tends to be more informative than frequentist approaches when mapping associations with small variance in outcome measures, while frequentist inference appears to be superior when associations with large variance are assessed^17^. In the present study, effect sizes of postoperative motor and non-motor symptom change were large, potentially contributing to the disparities in results and the pronounced associations of Bayesian factor mapping with clinical outcomes. Moreover, given that the NMSS is a composite score encompassing a wide variety of non-motor symptoms, the variance in postoperative symptom change is large, as observed in our sample. This could potentially limit the validity of employing the Bayes factor mapping approach in the present study. Nevertheless, the very strong evidence for h1 in specific clusters, including bilateral hippocampus and left insula could indicate a possible association of morphometry metrics with outcomes of specific non-motor symptoms such as memory and sensory processing.

However, effect sizes of postoperative motor and non-motor symptom change were similar, and we observed the aforementioned association between motor symptoms and brain morphometric features. Therefore, we reason that our findings for overall non-motor symptom burden genuinely reflect the absence of an effect rather than being attributed to low sensitivity. As non-motor symptoms constitute a heterogeneous group of symptoms^18,19^, several aspects have to be considered when interpreting the present findings.

First, this study investigated the brain areas associated with postoperative changes in a wide range of non-motor symptoms assessed by the NMSS total and its domain scores. Previous studies found limited evidence of an association between cortical atrophy and postoperative changes in various cognitive functions^20^. Lower hippocampal volume, however, has been associated with a postoperative decline in verbal memory^20,21^. In the present study, lower hippocampal volumes were associated with less postoperative changes in the attention and memory domains, although these results did not survive TFCE correction. The lack of significant results in this domain might be attributed to the presumably low sensitivity of the NMSS to detect subtle changes in a single cognitive domain. The NMSS is a clinician-rated scale investigating a wide range of NMS across nine domains whereby symptoms are rated according to severity and frequency. Thus, it is not intended to evaluate specific cognitive domains but global cognition and to assess the progress or treatment response of a wide range of non-motor symptoms^22^.

Second, the present study investigated the association between brain morphometry and short-term non-motor outcomes, not the development or worsening of pre-existing non-motor symptoms, which may result from the progression of Parkinson’s disease rather than from neurostimulation. In this context, Aybek and colleagues identified hippocampal volume as a marker to predict postsurgical conversion to dementia in the long-term, i.e., 25 months, follow-up^23^. Patients with Parkinson’s disease dementia had smaller preoperative hippocampal volumes than patients without conversion. The authors concluded that hippocampal atrophy is a potential clinical marker to predict postoperative conversion to dementia, but that the postsurgical development is due to the disease progression rather than the procedure itself^23^. As the present study investigated short-term outcomes only, further studies investigating the relationship between brain morphometry and long-term non-motor outcomes are needed.

Third, it is now widely accepted that the DBS effects are mediated via mechanisms on multiple levels, encompassing the micro- (e.g., local spiking activity), meso- (e.g., local field potentials), and macro-scale (e.g., interregional networks)^24^. In particular, network effects of DBS have received increasing attention in recent years, and it has been postulated that integrating a patient’s connectome into surgical planning could facilitate personalized DBS therapy^4^. Non-motor outcomes following STN-DBS depend on the location of neurostimulation^24^. Furthermore, previous studies have associated the stimulation of specific fiber tracts with postoperative outcomes such as depression and impulsivity^25,26^. In summary, neuromodulation of subcortical brain regions and connected functional brain networks is associated with non-motor outcomes, whereas morphometry metrics evident in routine MRI scans are not significantly associated with outcomes of overall non-motor burden. The clinical implication of our study is that volume loss in the superior frontal cortex should be considered as a potential MRI-based predictor of the subpar motor outcome of STN-DBS, whereas cortical volume loss does not indicate worse outcomes of overall non-motor burden. Future studies in this field should encompass multi-center designs involving a substantial patient cohort to systematically evaluate the associations between specific non-motor symptoms and morphometric metrics. Compared to these metrics of morphometry based on routine MRI examinations, it is conceivable that more advanced imaging techniques, e.g., markers of cerebral microstructure, such as neurite density, and connectivity measures are more sensitive to map non-motor treatment effects^27^.

This research has its limitations. First, despite being one of the largest cohorts of its kind, the sample size is relatively small. Nonetheless, it is unlikely that our sample was underpowered because the effect size of postoperative changes of motor and non-motor symptoms was comparable (Cohen’s *d*: 0.45, respectively 0.46), and we observed an association between metrics of morphometry and motor, but not non-motor outcomes. Second, we did not employ scales that specifically measure certain motor and non-motor symptoms, such as the Bain and Findley tremor scale for tremor, the Parkinson’s disease Sleep Scale (PDSS) for sleep, or the Montreal Cognitive Assessment (MoCA) for cognitive symptoms as we were interested in the association between brain morphometry and global motor and non-motor symptom burden. Therefore, we chose the UPDRS-III and the NMSS-T, which represent composite scores for motor and non-motor symptom severity. Moreover, our analysis was confined to VBM, potentially constraining generalizability. This choice, however, was made to remain concise and maintain consistency with previous studies that utilized UPDRS-III as an outcome parameter and VBM as the analysis method^10^. Third, the exact scanning parameters (e.g., repetition-time (TR) and echo-time (TE)) differed slightly across the sample. However, in a complementary analysis, we harmonized VBM metrics across scanners to overcome this limitation and received the same results. Fourth, the general limitations of VBM are inherent to our analysis as well. Despite being a powerful tool, VBM has several limitations including challenges with spatial normalization as well as co-registration that, together with partial volume effects, can potentially introduce systematic bias in the results. Furthermore, VBM provides information about structural properties in terms of intensity metrics but does not offer specific insights into histology^28^. However, the use of standardized processing pipelines implemented in open-source software, as employed in this study, can help to overcome the challenges named above and to reduce bias to a minimum. Fifth, in accordance with international guidelines, patients with cognitive impairment were excluded from DBS surgery. Consequently, and in line with other DBS studies, there were no further detailed exclusion criteria for cognitive impairment employed in the present study.

In conclusion, our study supports the importance of intact superior frontal cortex integrity as a predictor for motor outcomes in PD patients undergoing STN-DBS. Despite several advantages, including being based on scans implemented as a standard preoperative procedure, the short scanning time, and established pipelines in analysing and interpreting findings, our results indicate that the use of VBM as a measure to inform the patient selection and preoperative counseling is limited to motor effects and does not extend to effects of STN-DBS on the overall non-motor burden.

## Methods

### Participants

Patients were enrolled in this prospective, observational, ongoing study upon written informed consent in a single center (University Hospital Cologne). Clinical diagnosis of PD was based on the UK Brain Bank Criteria, and indication for DBS surgery was established according to international guidelines^29,30^. Study exclusion criteria comprised impaired visual and auditory function. The study was carried out following the Declaration of Helsinki and approved by the University of Cologne ethics committee (study no.: 12-145; German Clinical Trials Register: DRKS00006735).

### Clinical assessment

Clinical assessments were conducted at the preoperative baseline in the ON-medication state (MedON) and six months after DBS surgery in the ON-medication/ON-stimulation state (MedON/StimON). The reason to conduct assessments in the MedON as well as the MedON/StimON state was based on our utilization of the NMSS as one of our two primary outcome measures. As the NMSS assesses symptoms experienced over the past four weeks, in which participants have been in the Med-ON state (and the Med-ON/Stim-ON state), we aimed to avoid the artificial introduction of differences and thus potential bias between the UPDRS and NMSS assessments.

Standardized case report forms were used to collect demographic and clinical data on both study visits, including a comprehensive neuropsychological assessment which comprised the following scales:

1. The non-motor symptoms scale (NMSS) is a scale evaluated by clinicians that consists of 30 items, which assess nine domains of non-motor symptoms, including (1) cardiovascular, (2) sleep/fatigue, (3) mood/apathy, (4) perceptual problems/hallucinations, (5) attention/memory, (6) gastrointestinal tract, (7) urinary, (8) sexual function, and (9) miscellaneous. The miscellaneous category includes questions regarding pain, the ability to smell/taste, weight change, and excessive sweating. The NMSS has been frequently used in DBS studies for PD^31–33^. The score on this scale ranges from 0, indicating no impairment, to 360, indicating maximum impairment, while the symptoms are evaluated over the past four weeks^22,34^.
2. The PD Questionnaire (PDQ)-8 is a self-reported short form of the PDQ-39 that assesses eight dimensions of quality of life (QoL) in patients with PD. The PDQ-8 has been frequently used in DBS studies for PD^35–37^. The scale is reported as a summary index (SI) and ranges from 0, indicating no impairment, to 100, indicating maximum impairment^2,38,39^.
3. The Unified Parkinson’s Disease Rating Scale part III (UPDRS-III) is a clinician-rated scale evaluating motor symptom severity. The UPDRS-III ranges from 0 (no motor impairment) to 108 (maximum motor impairment)^40^.
4. The Scales for Outcomes in PD—motor function (SCOPA-M) is a scale evaluated by clinicians that assesses different dimensions of function in PD patients, including activities of daily living and motor complications. The subscale scores range from 0 (no impairment) to 21 for activities of daily living and 12 for motor complications^41^.

The levodopa equivalent daily dose (LEDD) was calculated based on the method described by Jost et al.^42^. Demographic and clinical characteristics are outlined in Table 1.

### MRI data acquisition

MRI acquisitions were performed on a 3T MRI system (Ingenia 3.0 T, Philips Healthcare or Achieva 3.0T, Philips Healthcare) in a single center (Cologne). Each PD patient in the MedON underwent a 3D T1-weighted magnetization prepared—rapid gradient echo sequence (MPRAGE) at baseline (for scanning parameters, see Supplementary Table 3).

At the time of image acquisition, images were investigated to be free of motion or ghosting and high frequency or wrap-around artefacts.

### Image processing

Voxel-based morphometry was performed within the Computational Anatomy Toolbox (CAT) analysis suite (CAT12.8.2, University Hospital Jena, Jena, Germany)^43^ implemented in statistical parametric mapping 12 (SPM12, Wellcome Department of Cognitive Neurology, London, United Kingdom). All steps were conducted in MATLAB R2022a (The MathWorks Inc., Natick, MA, USA), as reported previously by Jergas et al.^44^. In short, processing included spatial registration to a template brain, segmentation into cortical gray matter, white matter, and cerebrospinal fluid, calculation of total intracranial volume (TIV), and empirical quality control (QC) using default parameters. QC was performed within the QC framework of CAT12 with scans not rating lower than B-. Finally, data smoothing was performed using an 8 mm full-width half-maximum isotropic Gaussian kernel. In a complementary analysis, we used ComBat to harmonize VBM metrics across scanners used in the present study^45^.

### Statistical analysis

Statistical analysis of clinical outcomes was performed using MATLAB R2018b. We employed the Shapiro–Wilk test to assess the assumption of normality. Subsequently, Wilcoxon signed-rank- or *t*-tests, when parametric test criteria were fulfilled, were employed to analyse changes between baseline and 6-month follow-up. The Benjamini–Hochberg method was used to control the false discovery rate, and effect sizes were calculated according to Cohen^46,47^. Reported *p* values are two-sided and were accepted as significant where *p* < 0.05.

Statistical voxelwise analysis of image data was performed using SPM12. Here, clinical outcomes were represented as change scores in UPDRS-III, NMSS-T, and NMSS-Domains and calculated according to the following Eq. (1):

1 $${{{\mathrm{Scale}}}}_{{{\mathrm{baseline}}}}-{{{\mathrm{Scale}}}}_{{{\mathrm{follow}}}-{{\mathrm{up}}}}$$

The decision to employ change scores, as opposed to percentage differences, was informed by the presence of small values for some of the NMSS-domains, as small values can lead to an overestimation of effects when employing percentage differences. Associations between surrogates of brain morphometry and motor and non-motor outcomes were assessed using a multiple regression analysis with age, sex, and total intracranial volume as covariates. We repeated the multiple regression analysis on the harmonized data using a stepwise approach where we included an ANCOVA to account for baseline inhomogeneity in the first step and incorporated disease duration as an additional covariate in the second step. A threshold-free cluster enhancement (TFCE) was applied to correct for multiple comparisons as implemented in the TFCE Toolbox. Results were accepted as significant where family-wise error-corrected *p* < 0.05.

In a complementary analysis, we performed voxelwise mapping of Bayes factor by Bayesian general linear models for both motor and non-motor outcomes. This analysis was performed within the framework of the BLDI toolbox implemented in R using sex and disease duration as covariates and employing VBM metrics harmonized across scanners^17^. For concise visualization, we present clusters with very strong evidence for H1 (log Bayes factor >1.48) in Supplementary Figs. S2, S3 as well as unthresholded clusters in Supplementary Figs. S4, S5.

### Reporting summary

Further information on research design is available in the Nature Research Reporting Summary linked to this article.

### Supplementary information

Supplementary Material

Reporting Summary

## Data Availability

The data supporting this study’s findings are available on reasonable request from the corresponding authors (PAL, HSD). The data were not publicly available due to privacy or ethical restrictions.

## Code availability

All tools used to analyse MRI data were based on CAT12 (https://neuro-jena.github.io/cat/), SPM12 (http://www.fil.ion.ucl.ac.uk/spm), ComBat (https://github.com/Jfortin1/ ComBatHarmonization), and the BLDI toolbox (https://github.com/ChrisSperber/BLDI).
