# Supplementary material for: No evidence for an association of voxel-based morphometry with short-term non-motor outcomes in deep brain stimulation for Parkinson’s disease
Source: NPJ Parkinsons Dis. 2024 Apr 26;10:91. doi: 10.1038/s41531-024-00695-1 (PMC11053137; doi:10.1038/s41531-024-00695-1)
Supplement: Supplementary file 1 — Supplementary Material [file 41531_2024_695_MOESM1_ESM.pdf]

Supplementary material to

"No evidence for an association of voxel-based morphometry with short-term non-motor outcomes in deep brain stimulation for Parkinson's disease"

Supplementary Table 1: Association between brain morphometry and UPDRS-III change after harmonization and inclusion of ANCOVA

Cluster	Location	p-Value	Cluster size	MNI152-Coordinates		
				X	Y	Z
1	Left superior frontal cortex	.003	1102	-14	50	18
2	Right superior frontal cortex	.029	226	12	59	15
3	Left orbitofrontal cortex	.032	113	8	50	-17

Supplementary Table 1. Characteristics of clusters with an association between metrics of voxel-based morphometry and postoperative change in UPDRS-III after data harmonization using ComBat and inclusion of ANCOVA to correct for baseline inhomogeneity. "Cluster" denotes clusters with a significant association between low cortical volumes and poor motor response to deep brain stimulation (DBS). "Location" indicates the anatomical landmark comprising the majority of voxels of a cluster, according to the Desikan-Killiany Atlas. P-Values are clusterwise p-values corrected for multiple comparisons. Cluster size denotes the extent of a cluster in voxel. "MNI152-coordinates" describe the coordinates of the cluster's center of gravity in MNI152-space.

Supplementary Table 2: Association between brain morphometry and UPDRS-III change after harmonization and inclusion of ANCOVA and disease duration

Cluster	Location	p-Value	Cluster size	MNI152-Coordinates		
				X	Y	Z
1	Left superior frontal cortex	.005	1311	-14	50	18
2	Right superior frontal cortex	.054	317	12	59	15

Supplementary Table 2. Characteristics of clusters with an association between metrics of voxel-based morphometry and postoperative change in UPDRS-III after data harmonization using ComBat and inclusion of ANCOVA to correct for baseline inhomogeneity and disease duration as an additional covariate. "Cluster" denotes clusters with a significant association between low cortical volumes and poor motor response to deep brain stimulation (DBS). "Location" indicates the anatomical landmark comprising the majority of voxels of a cluster, according to the Desikan-Killiany Atlas. P-Values are clusterwise p-values corrected for multiple comparisons. Cluster size denotes the extent of a cluster in voxel. "MNI152-coordinates" describe the coordinates of the cluster's center of gravity in MNI152-space.

Supplementary Table 3: Imaging parameters for individual participants

Subject	Repetition Time	Echo Time	Scanner	Slices	Flip Angle	Field of view	Slice Thickness	Acquisition Matrix
1	9.602	4.784	Ingenia	174	8	250	2	[0;252;250;0]
2	9.580	4.766	Achieva	176	8	250	2	[0;252;250;0]
3	9.608	4.779	Achieva	172	8	250	2	[0;252;250;0]
4	9.573	4.774	Achieva	167	8	250	2	[0;252;250;0]
5	9.609	4.798	Achieva	168	8	250	2	[0;252;250;0]
6	9.664	4.788	Achieva	165	8	250	2	[0;252;250;0]
7	9.472	4.728	Achieva	162	8	250	2	[0;252;250;0]
8	9.397	4.698	Achieva	165	8	250	2	[0;252;250;0]
9	9.467	4.713	Achieva	173	8	250	2	[0;252;250;0]
10	9.717	4.854	Ingenia	167	8	250	2	[0;252;250;0]
11	9.666	4.775	Achieva	167	8	250	2	[0;252;250;0]
12	9.673	4.837	Ingenia	165	8	250	2	[0;252;250;0]
13	9.509	4.747	Achieva	173	8	250	2	[0;252;250;0]
14	9.729	4.844	Ingenia	167	8	250	2	[0;252;249;0]
15	9.816	4.877	Ingenia	167	8	250	2	[0;252;249;0]
16	9.624	4.77	Achieva	167	8	250	2	[0;252;250;0]
17	9.876	4.865	Ingenia	167	8	250	2	[0;252;250;0]
18	9.870	4.913	Ingenia	180	8	250	2	[0;252;250;0]
19	9.645	4.793	Achieva	168	8	250	2	[0;252;250;0]
20	9.739	4.857	Ingenia	167	8	250	2	[0;252;250;0]
21	9.939	4.929	Ingenia	175	8	250	2	[0;252;250;0]
22	9.530	4.736	Achieva	167	8	250	2	[0;252;250;0]
23	9.443	4.714	Achieva	167	8	250	2	[0;252;250;0]
24	9.648	4.79	Achieva	177	8	250	2	[0;252;250;0]
25	9.468	4.724	Achieva	167	8	250	2	[0;252;250;0]
26	9.757	4.844	Ingenia	167	8	250	2	[0;252;250;0]
27	9.882	4.882	Ingenia	167	8	250	2	[0;252;250;0]
28	9.530	4.76	Ingenia	167	8	250	2	[0;252;249;0]
29	9.655	4.819	Ingenia	167	8	250	2	[0;252;249;0]
30	9.778	4.867	Ingenia	182	8	250	2	[0;252;250;0]
31	9.863	4.893	Ingenia	167	8	250	2	[0;252;250;0]
32	9.502	4.735	Achieva	167	8	250	2	[0;252;250;0]
33	9.639	4.818	Ingenia	167	8	250	2	[0;252;250;0]
34	9.667	4.828	Ingenia	167	8	250	2	[0;252;250;0]
35	9.702	4.852	Ingenia	167	8	250	2	[0;252;249;0]
36	9.615	4.803	Achieva	165	8	250	2	[0;252;250;0]
37	9.719	4.849	Ingenia	167	8	250	2	[0;252;250;0]
38	9.821	4.852	Ingenia	167	8	250	2	[0;252;250;0]
39	9.612	4.759	Achieva	178	8	250	2	[0;252;250;0]
40	9.568	4.767	Achieva	167	8	250	2	[0;252;250;0]
41	9.590	4.788	Ingenia	167	8	250	2	[0;252;250;0]
42	9.847	4.914	Ingenia	165	8	250	2	[0;252;250;0]
43	9.702	4.815	Achieva	177	8	250	2	[0;252;250;0]
44	9.625	4.777	Achieva	173	8	250	2	[0;252;250;0]
45	9.515	4.748	Achieva	168	8	250	2	[0;252;250;0]
46	9.628	4.8	Achieva	168	8	250	2	[0;252;250;0]
47	9.763	4.843	Ingenia	175	8	250	2	[0;252;250;0]
48	9.396	4.694	Achieva	168	8	250	2	[0;252;250;0]
49	9.795	4.868	Ingenia	181	8	250	2	[0;252;250;0]

Supplementary Figure 1: Association of voxel-based morphometry metrics and postoperative motor symptom change using a multiple regression analysis

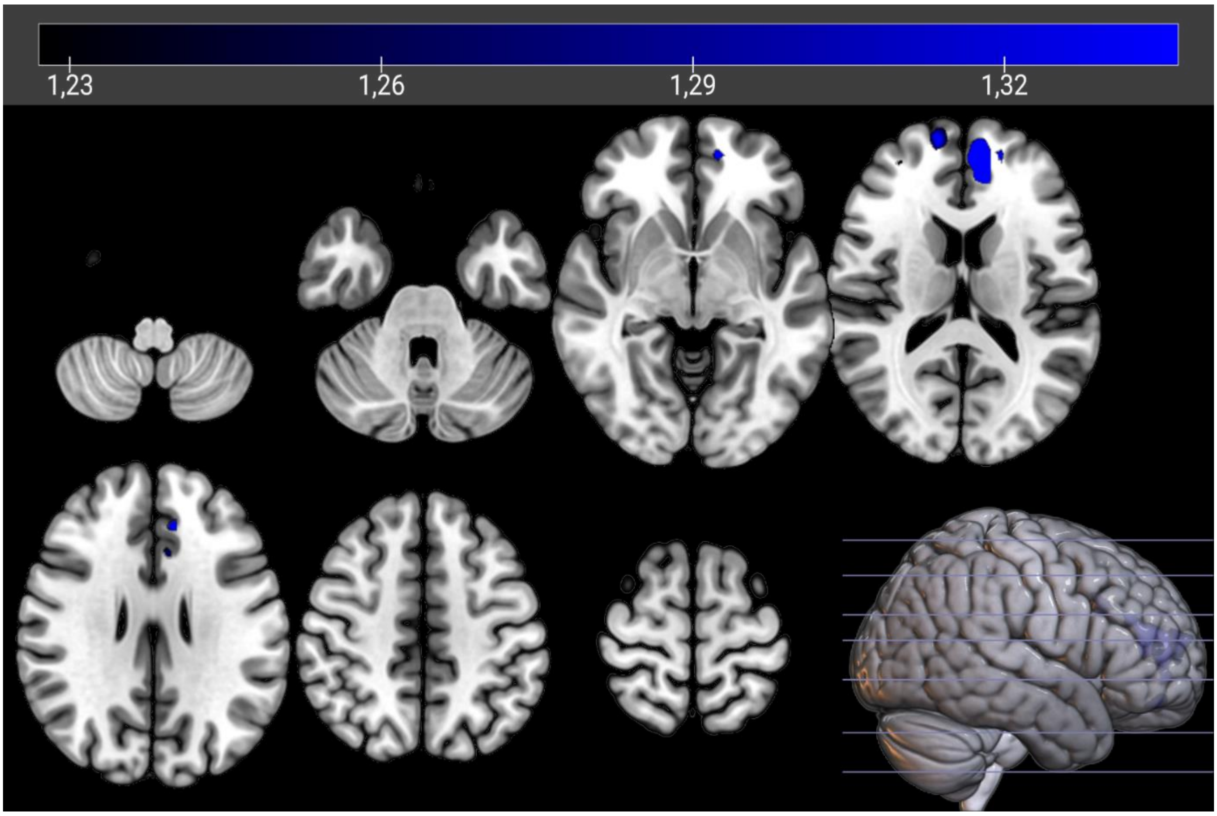

Supplementary Figure 1. Clusters with an association between metrics of voxel-based morphometry and postoperative change in UPDRS-III as revealed by the whole brain analysis. Results are displayed as overlays. Clusters denote regions of low cortical volumes significantly associated with poor motor response to deep brain stimulation. P-Values were corrected for multiple comparisons using a threshold-free cluster enhancement (TFCE) approach and thresholded at p<.05, family-wise error-corrected. The color bar indicates TFCE values.

Supplementary Figure 2: Results of Bayesian factor mapping for UPDRS-III change

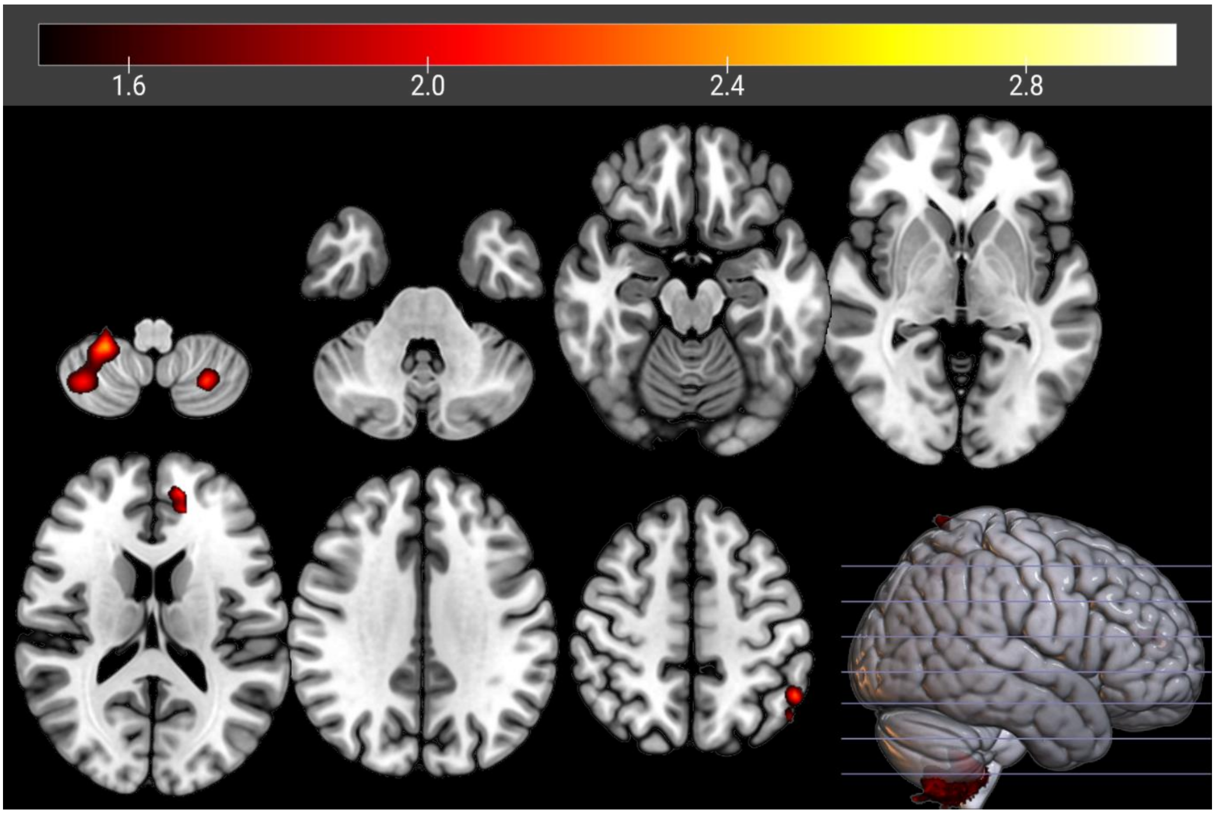

Supplementary Figure 2. Voxel wise mapping of Bayes factor by Bayesian general linear modelling for motor outcomes using harmonized scans. Maps were thresholded at a log Bayes factor of 1.48 to depict clusters with very strong evidence for an association between brain morphometry and non-motor outcomes. Results are displayed as overlays.

Supplementary Figure 3: Results of Bayesian factor mapping for NMSS-T change

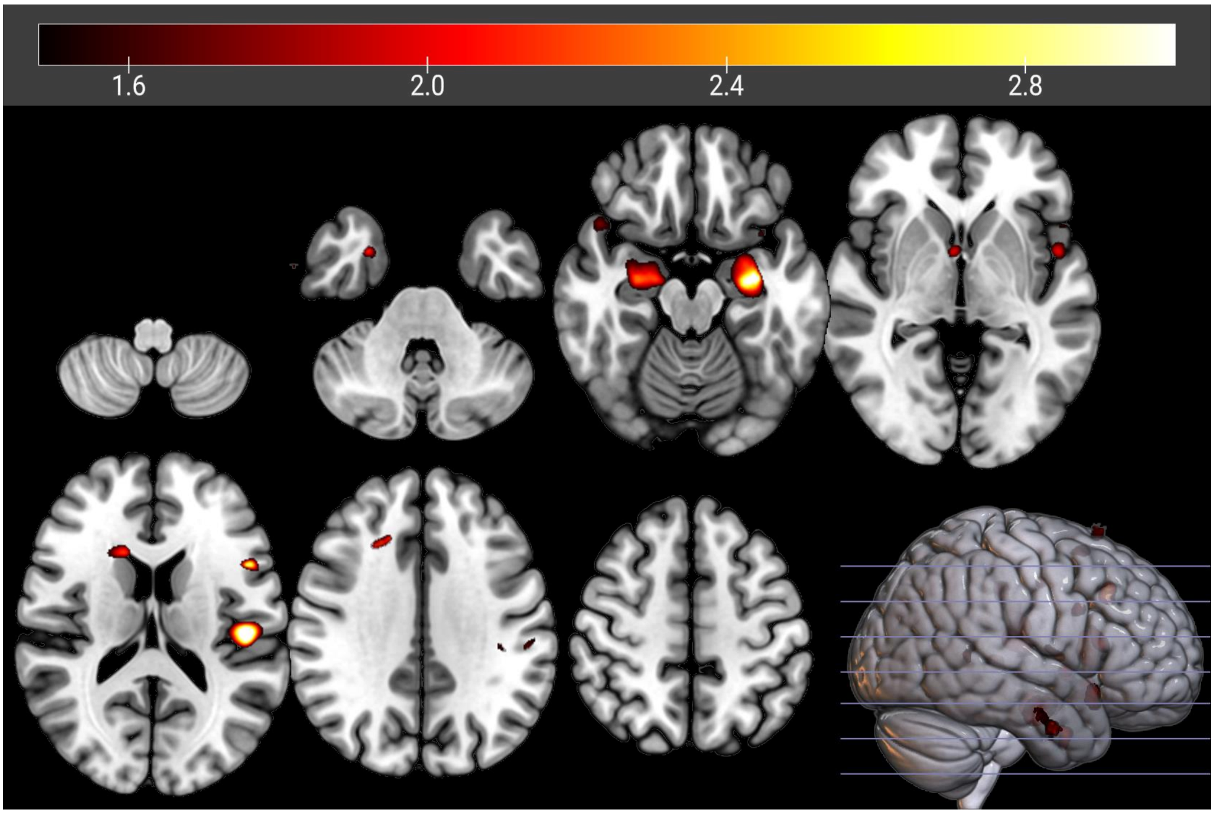

Supplementary Figure 3. Voxel wise mapping of Bayes factor by Bayesian general linear modelling for changes in non-motor burden using harmonized scans. Maps were thresholded at a log Bayes factor of 1.48 to depict clusters with very strong evidence for an association between brain morphometry and non-motor outcomes. Results are displayed as overlays.

Supplementary Figure 4: Results of Bayesian factor mapping for UPDRS-III change

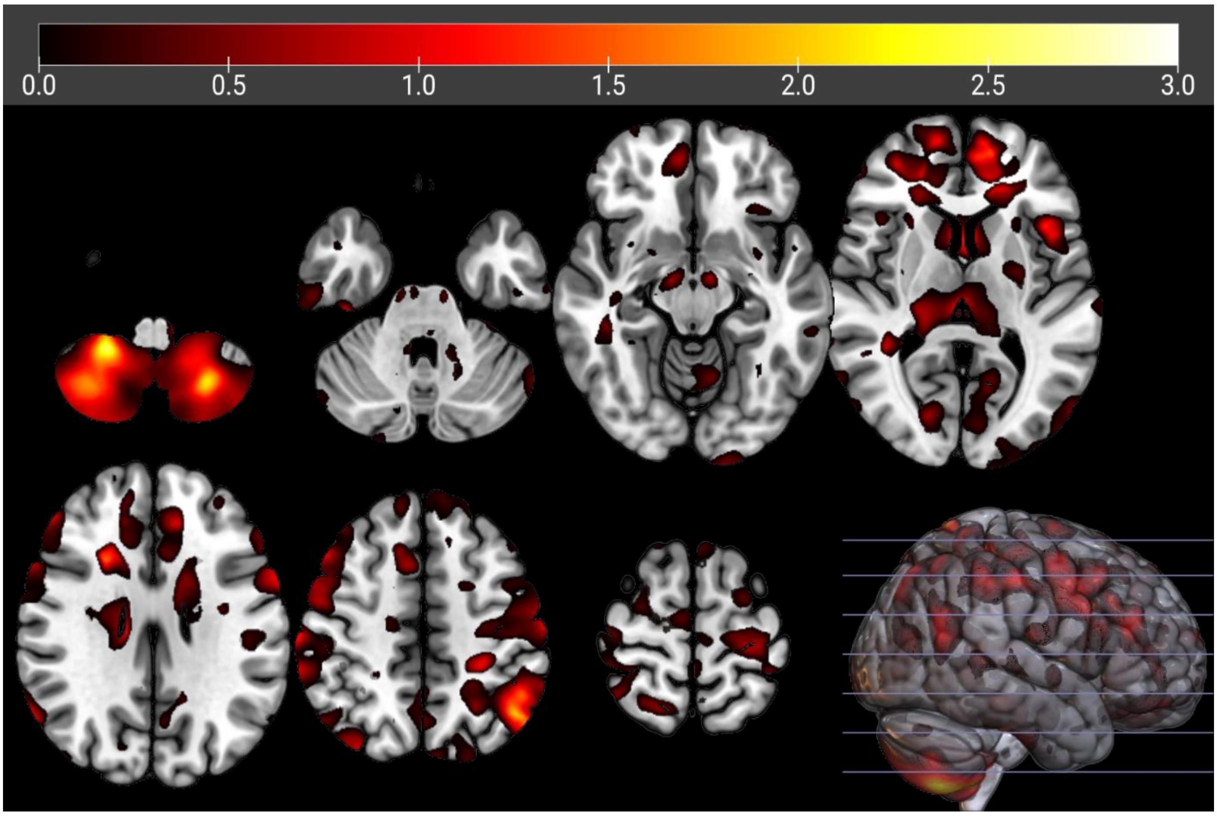

Supplementary Figure 4. Voxel wise mapping of Bayes factor by Bayesian general linear modelling for motor outcomes using harmonized scans. Maps are unthresholded and displayed as overlays.

Supplementary Figure 5: Results of Bayesian factor mapping for NMSS-T change

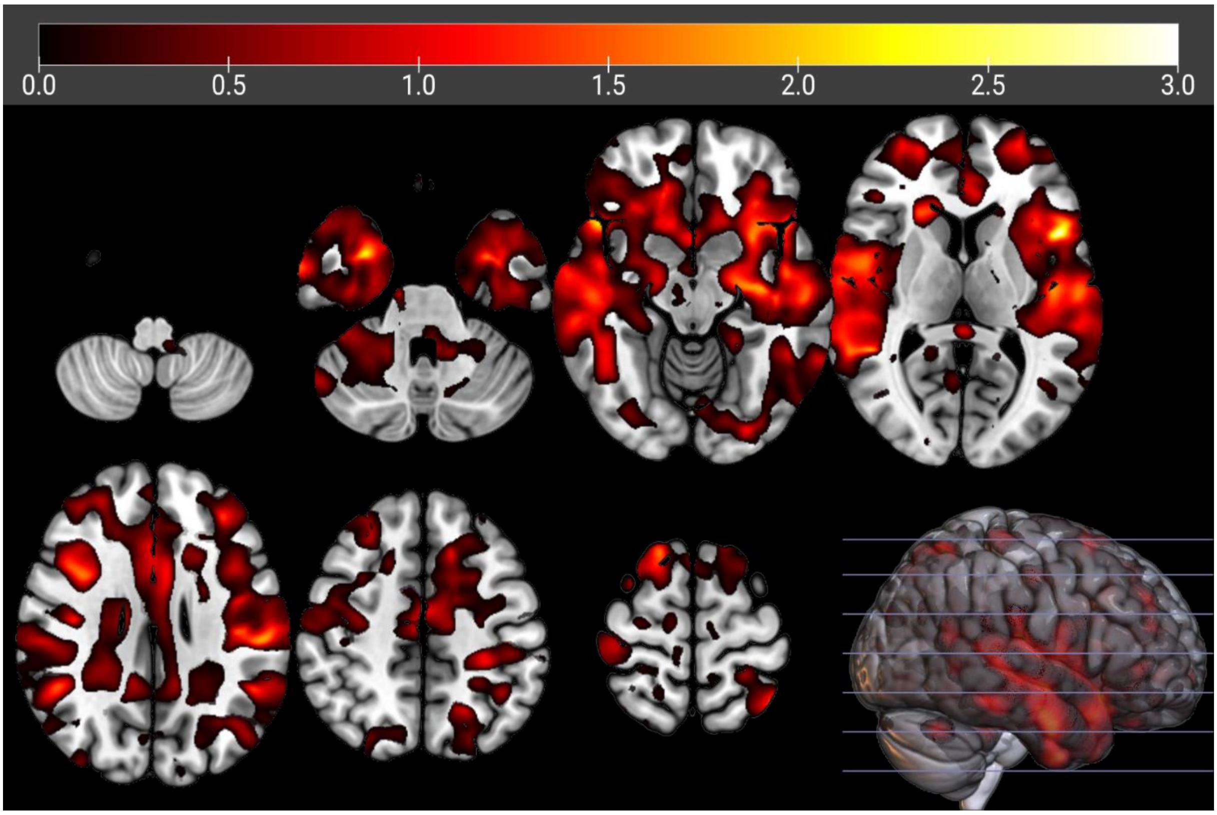

Supplementary Figure 5. Voxel wise mapping of Bayes factor by Bayesian general linear modelling for changes in non-motor burden using harmonized scans. Maps are unthresholded and displayed as overlays.

Supplementary Figure 6: Linear Relationship between UPDRS-III improvement and Morphometry

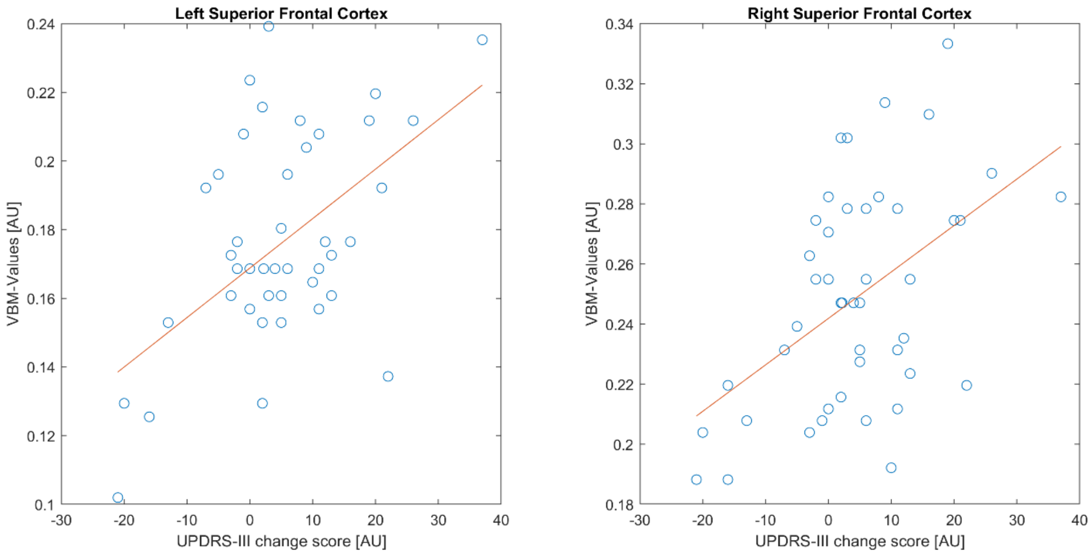

Supplementary Figure 6. Scatter plots depicting the relationship between UPDRS-III change scores and VBM-values.

Non-Motor Parkinson's Disease Study Group

Members of the MDS Non-Motor Parkinson's Disease Study Group listed here did not contribute to the current study.

Adler, Charles[15]
Bhidayasiri, Roongroj[16]
Borghammer, Per[17]
Barone, Paolo[18]
Brooks, David J.[19]
Brown, Richard[20]
Cantillon, Marc[21]
Carroll, Camille[22]
Coelho, Miguel[23]
Falup-Pecurariu, Cristian[24]
Henriksen, Tove[25]
Hu, Michele[26]
Jenner, Peter[27]
Jeon, Beomseok[28]
Kramberger, Milica[29]
Kumar, Padma[30]
Kurtis, Mónica[31]

Leta, Valentina[9]
Lewis, Simon[32]
Litvan, Irene[33]
Lyons, Kelly[34]
Martino, Davide[35]
Masellis, Mario[36]
Mochizuki, Hideki[37]
Morley, James F.[38]
Nirenberg, Melissa[39]
Odin, Per[40]
Pagonabarraga, Javier[41]
Panicker, Jalesh[42]
Pavese, Nicola[43]
Pekkonen, Eero[44]
Postuma, Ron[45]
Rodriguez Violante, Mayela[46]
Rosales, Raymond[47]
Schapira, Anthony[48]
Schrag, Anette[49]
Simuni, Tanya[50]
Stocchi, Fabrizio[51]
Storch, Alexander[52]
Subramanian, Indu[53]
Tagliati, Michele[54]
Tinazzi, Michele[55]
Toledo, Jon[56]
Tsuboi, Yoshio[57]
Walker, Richard[58]
Weintraub, Daniel[59]

[15] The Parkinson's Disease and Movement Disorders Center, Department of Neurology, Mayo Clinic, Scottsdale, Arizona, USA
[16]Chulalongkorn Centre of Excellence for Parkinson's Disease & Related Disorders, Department of Medicine, Faculty of Medicine, Chulalongkorn University and King Chulalongkorn Memorial Hospital, Thai Red Cross Society, Bangkok, Thailand
[17] Nuclear Medicine and PET, Aarhus University Hospital, Aarhus, Denmark
[18] Center for Neurodegenerative Diseases (CEMAND), Neuroscience Section, University of Salerno, Salerno, Italy
[19] Institute of Neuroscience, Newcastle University, Newcastle, UK; Department of Nuclear Medicine and PET Centre, Aarhus University Hospital, Aarhus, Denmark
[20] King's College London, Department of Psychology, London, UK
[21] Reviva Pharmaceuticals, Inc., Santa Clara, CA, USA
[22] Faculty of Medicine and Dentistry, University of Plymouth, Plymouth, UK
[23] FAS Center for Systems Biology, Harvard University, Cambridge, MA, USA
[24] Faculty of Medicine, Transilvania University of Brașov, Brașov, Romania
[25] Movement Disorder Clinic, University Hospital of Bispebjerg, Copenhagen, NV, Denmark
[26] Oxford Parkinson's Disease Centre, University of Oxford, UK; Nuffield Department of Clinical Neurosciences, University of Oxford, UK
[27] Neurodegenerative Diseases Research Group, Institute of Pharmaceutical Sciences, Faculty of Life Sciences and Medicine, King's College London, Newcomen Street, London, UK
[28] Department of Neurology, Seoul National University College of Medicine, Seoul, South Korea

[29] Division of Clinical Geriatrics, Department of Neurobiology, Care Sciences and Society, Center for Alzheimer Research, Karolinska Institutet, Stockholm, Sweden Department of Neurology, University Medical Centre Ljubljana, Ljubljana, Slovenia
[30] Parkinson's Disease Service for the Older Person, Rankin Park Centre, John Hunter Hospital, HNELHD, Newcastle, NSW, Australia
[31] Functional Movement Disorders Unit, Movement Disorders Program, Neurology Department, Hospital Ruber Internacional, Madrid, Spain
[32] Brain and Mind Centre, University of Sydney, NSW, Australia
[33] Department of Neurosciences Movement Disorders Center, University of California, San Diego, USA
[34] University of Kansas Medical Center, Kansas City, KS, USA
[35] Department of Clinical Neurosciences, University of Calgary & Hotchkiss Brain Institute, Calgary, Canada
[36] Hurvitz Brain Sciences Program, Sunnybrook Research Institute, Toronto, ON, Canada
[37] Department of Neurology, Osaka University Graduate School of Medicine, Osaka, Japan
[38] Parkinson Disease Research, Education, and Clinical Center, Philadelphia Veteran Affairs Medical Center, Philadelphia, PA, USA; Department of Neurology, University of Pennsylvania, Philadelphia, PA, USA
[39] Department of Neurology, NYU School of Medicine, New York, NY, USA
[40] University of Lund, Faculty of Medicine, Lund, Sweden
[41] Movement Disorders Unit, Sant Pau Hospital and Biomedical Research Institute (IIB-Sant Pau), Barcelona, Spain
[42] Neurology, National Hospital for Neurology & Neurosurgery, London, United Kingdom
[43] Newcastle Magnetic Resonance Centre & Positron Emission Tomography Centre, Newcastle University, Campus for Ageing & Vitality, Newcastle upon Tyne, United Kingdom
[44] Department of Neurology, Helsinki University Hospital, and Department of Neurological Sciences (Neurology), University of Helsinki, Helsinki, Finland
[45] Research Institute of McGill University Health Centre, Montréal, Canada
[46] Movement Disorders Clinic, National Institute of Neurology and Neurosurgery, Mexico City, Mexico
[47] Department of Neurology and Psychiatry, University of Santo Tomas Hospital, Manila 1008, Philippines; International Institute of Neuroscience, Saint Luke's Medical Center, Philippines; Center for Neurodiagnostic and Therapeutic Services, Metropolitan Medical Center, Manila 1000, Philippines
[48] Department of Clinical Neurosciences, University College London (UCL) Institute of Neurology, Royal Free Campus, Rowland Hill Street, London, UK
[49]UCL Institute of Neurology, London, United Kingdom
[50] Department of Neurology, Northwestern University, Feinberg School of Medicine, Chicago, IL , USA
[51] University and Institute for Research and Medical Care, IRCCS San Raffaele, Rome, Italy
[52] Division of Neurodegenerative Diseases, Department of Neurology, Dresden University of Technology, Dresden, Germany Department of Neurology, Dresden University of Technology, Dresden, Germany German Center for Neurodegenerative Diseases (DZNE), Research Site Dresden, Dresden, Germany
[53] UCLA/West LA VA, Los Angeles, CA, United States
[54] Cedars-Sinai Medical Center, Los Angeles, CA, United States
[55] Department of Neuroscience, Biomedicine, and Movement, University of Verona, Verona, Italy
[56] Department of Pathology & Laboratory Medicine, University of Pennsylvania, Philadelphia, PA, USA; Department of Neurology, Houston Methodist Hospital, Houston, TX, USA
[57] Department of Neurology, Fukuoka University, Japan
[58] Northumbria Healthcare NHS Foundation Trust, North Tyneside General Hospital, Rake Lane, North Shields, Tyne and Wear, United Kingdom
[59] Department of Psychiatry and Department of Neurology, University of Pennsylvania School of Medicine, Philadelphia, PA, USA; Parkinson's Disease and Mental Illness Research, Education and Clinical Centers, Philadelphia Veterans Affairs Medical Center, Philadelphia, PA, USA
