# Supplementary material for: No evidence for an association of voxel-based morphometry with short-term non-motor outcomes in deep brain stimulation for Parkinson’s disease
Source: NPJ Parkinsons Dis. 2024 Apr 26;10:91. doi: 10.1038/s41531-024-00695-1 (PMC11053137; doi:10.1038/s41531-024-00695-1)
Supplement: Supplementary file 2 — Reporting Summary [file 41531_2024_695_MOESM2_ESM.pdf]

Corresponding author(s): Philipp Loehrer, Haidar Dafsari
Last updated by author(s): Oct 25, 2023

Reporting Summary

Nature Portfolio wishes to improve the reproducibility of the work that we publish. This form provides structure for consistency and transparency in reporting. For further information on Nature Portfolio policies, see our Editorial Policies and the Editorial Policy Checklist.

Statistics

For all statistical analyses, confirm that the following items are present in the figure legend, table legend, main text, or Methods section.

n/a	Confirmed	
☐	☒	The exact sample size (n) for each experimental group/condition, given as a discrete number and unit of measurement
☐	☒	A statement on whether measurements were taken from distinct samples or whether the same sample was measured repeatedly
☐	☒	The statistical test(s) used AND whether they are one- or two-sided<br>*Only common tests should be described solely by name; describe more complex techniques in the Methods section.*
☐	☒	A description of all covariates tested
☐	☒	A description of any assumptions or corrections, such as tests of normality and adjustment for multiple comparisons
☐	☒	A full description of the statistical parameters including central tendency (e.g. means) or other basic estimates (e.g. regression coefficient) AND variation (e.g. standard deviation) or associated estimates of uncertainty (e.g. confidence intervals)
☐	☒	For null hypothesis testing, the test statistic (e.g. F, t, r) with confidence intervals, effect sizes, degrees of freedom and P value noted<br>*Give P values as exact values whenever suitable.*
☐	☒	For Bayesian analysis, information on the choice of priors and Markov chain Monte Carlo settings
☒	☐	For hierarchical and complex designs, identification of the appropriate level for tests and full reporting of outcomes
☐	☒	Estimates of effect sizes (e.g. Cohen's d, Pearson's r), indicating how they were calculated

Our web collection on statistics for biologists contains articles on many of the points above.

Software and code

Policy information about availability of computer code

Data collection	No software was used for collection of clinical data.
Data analysis	All tools used to analyse MRI data are based on CAT12 (https://neuro-jena.github.io/cat/), SPM12 (http://www.fil.ion.ucl.ac.uk/spm), ComBat (https://github.com/Jfortin1/ComBatHarmonization), and the BLDI toolbox (https://github.com/ChrisSperber/BLDI).

For manuscripts utilizing custom algorithms or software that are central to the research but not yet described in published literature, software must be made available to editors and reviewers. We strongly encourage code deposition in a community repository (e.g. GitHub). See the Nature Portfolio guidelines for submitting code & software for further information.

Data

Policy information about availability of data

All manuscripts must include a data availability statement. This statement should provide the following information, where applicable:

- Accession codes, unique identifiers, or web links for publicly available datasets
- A description of any restrictions on data availability
- For clinical datasets or third party data, please ensure that the statement adheres to our policy

The data supporting this study’s findings are available on reasonable request from the corresponding authors (PAL, HSD). The data are not publicly available due to privacy or ethical restrictions.

Research involving human participants, their data, or biological material

Policy information about studies with human participants or human data. See also policy information about sex, gender (identity/presentation), and sexual orientation and race, ethnicity and racism.

Reporting on sex and gender	In the present study, sex was determined based on self-reporting. As this is an ongoing open-label study recruiting patients that undergo deep brain stimulation of the subthalamic nucleus for Parkinson's disease, neither sex nor gender were considered in the study design. Overall, 18 female (36.7%) and 31 male (63.3%) patients were assessed in this analysis. Sex was used as a covariate for regression analyses as well as Bayes factor mapping.
Reporting on race, ethnicity, or other socially relevant groupings	We did not obtain information on race, ethnicity or other socially constructed categories.
Population characteristics	Covariate-relevant population characteristics were age, sex, disease duration, and total intracranial volume.
Recruitment	Participants were recruited via the outpatient clinic of the University Hospital Cologne (Department of Neurology).
Ethics oversight	The study was carried out following the Declaration of Helsinki and approved by the University of Cologne ethics committee (study no.: 12-145)

Note that full information on the approval of the study protocol must also be provided in the manuscript.

Field-specific reporting

Please select the one below that is the best fit for your research. If you are not sure, read the appropriate sections before making your selection.

☒ Life sciences ☐ Behavioural & social sciences ☐ Ecological, evolutionary & environmental sciences

For a reference copy of the document with all sections, see nature.com/documents/nr-reporting-summary-flat.pdf

Life sciences study design

All studies must disclose on these points even when the disclosure is negative.

Sample size	Based on the general trend in the field of VBM analysis as well as our experience, a sample size of 49 subjects should give meaningful and reasonable results (cf. https://doi.org/10.1016/j.neuroimage.2020.116982 or https://doi.org/10.1016/j.neuroimage.2019.116344 for recent publications). We used data from 49 subjects for the final analysis. Details are given in the Methods section.
Data exclusions	No data was excluded for final analysis of the association between VBM metrics and NMSS-T (and subscore) values. Four patients were excluded for the analysis of the association between VBM metrics and UPDRS-III values, as they did not complete this assessment during follow-up for various reasons.
Replication	In this study, a large set of participants was analyzed (N=49). We replicated our findings using different approaches (Combat, TFCE with ANCOVA, without ANCOVA).
Randomization	Randomization was not relevant for this trial as we investigated the association between metrics of voxel-based morphology with postoperative motor and non-motor outcomes.
Blinding	No pharmacological or comparable intervention took place. Furthermore, de-identified data was used during analysis steps. Therefore, blinding was not relevant for this study.

Reporting for specific materials, systems and methods

We require information from authors about some types of materials, experimental systems and methods used in many studies. Here, indicate whether each material, system or method listed is relevant to your study. If you are not sure if a list item applies to your research, read the appropriate section before selecting a response.

Materials & experimental systems

n/a	Involved in the study
☒	☐ Antibodies
☒	☐ Eukaryotic cell lines
☒	☐ Palaeontology and archaeology
☒	☐ Animals and other organisms
☐	☒ Clinical data
☒	☐ Dual use research of concern
☒	☐ Plants

Methods

n/a	Involved in the study
☒	☐ ChIP-seq
☒	☐ Flow cytometry
☐	☒ MRI-based neuroimaging

Clinical data

Policy information about clinical studies

All manuscripts should comply with the ICMJE guidelines for publication of clinical research and a completed CONSORT checklist must be included with all submissions.

Clinical trial registration	German Clinical Trials Register: DRKS00006735
Study protocol	https://drks.de/search/en/trial/DRKS00006735
Data collection	Data was collected during study visits prior to and 6 months after deep brain stimulation. The MRI data was obtained during the preoperative assessment. All data was collected in a single center (University Hospital Cologne)
Outcomes	UPDRS-III and NMSS-T outcomes were defined as primary outcome measures based on the existing VBM-STN-DBS literature in PD (for UPDRS-III; to compare our results with the existing literature) and as there were no prior studies assessing VBM and NMS in PD (for NMSS).

Plants

Seed stocks	-
Novel plant genotypes	-
Authentication	-

Magnetic resonance imaging

Experimental design

Design type	Structural MRI, resting state.
Design specifications	NA
Behavioral performance measures	NA

Acquisition

Imaging type(s)	structural
Field strength	3T
Sequence & imaging parameters	Please see supplementary table S3
Area of acquisition	whole brain
Diffusion MRI	☐ Used ☒ Not used

Preprocessing

Preprocessing software	Computational Anatomy Toolbox (CAT) analysis suite (CAT12.8.2, University Hospital Jena, Jena, Germany)
Normalization	For spatial registration and normalization, CAT uses Geodesic Shooting (Ashburner and Friston, 2011) with predefined templates (c.f. CAT12 manual).
Normalization template	ICBM 2009c Nonlinear Asymmetric space (MNI152NLin2009cAsym; https://www.bic.mni.mcgill.ca/ServicesAtlases/ICBM152NLin2009),
Noise and artifact removal	The study's processing pipeline employed the steps included in the CAT12 analysis suit for voxel-based processing using default parameters. This included applying a spatial adaptive non-local means (SANLM) denoising filter, resampling, bias-correction, affine-registration, and segmentation. For further details please c.f. https://neuro-jena.github.io/cat12-help/#process_details
Volume censoring	Final results are displayed using a threshold of p = .05 for the frequentist statistics, whereas maps were thresholded at a log Bayes factor of 1.48 to depict clusters with very strong evidence for h1 for Bayes factor mapping.

Statistical modeling & inference

Model type and settings	Multiple regression analysis
Effect(s) tested	Associations between surrogates of brain morphometry and motor and non-motor outcomes were assessed using a multiple regression analysis with age, sex, and total intracranial volume as covariates.

Specify type of analysis: ☒ Whole brain ☐ ROI-based ☐ Both

Statistic type for inference (See Eklund et al. 2016)	voxel-wise
Correction	A threshold-free cluster enhancement (TFCE) was applied to correct for multiple comparisons as implemented in the TFCE Toolbox.

Models & analysis

n/a	Involved in the study
☒	☐ Functional and/or effective connectivity
☒	☐ Graph analysis
☒	☐ Multivariate modeling or predictive analysis
